# The relationship between employment status and depression symptomatology among women at risk for postpartum depression

**DOI:** 10.1177/1745505717708475

**Published:** 2017-05-07

**Authors:** Beth A Lewis, Lauren Billing, Katie Schuver, Dwenda Gjerdingen, Melissa Avery, Bess H Marcus

**Affiliations:** 1 University of Minnesota, Minneapolis, MN, USA; 2 University of California, San Diego, La Jolla, CA, USA

**Keywords:** employment status, exercise, physical activity, postpartum depression, women’s health

## Abstract

Approximately 13%–19% of new mothers report depression during the postpartum period. Returning to work after childbirth is associated with depression; however, it is unclear if this finding applies to women who are at high risk for postpartum depression. The purpose of this study was to examine the relationship between employment status and depression symptomatology among women at risk for postpartum depression (defined as personal or maternal history of depression). This study was a post hoc analysis from a previously conducted randomized controlled trial. Participants (n = 124; ages 18–42) were 7 months postpartum and had participated in a randomized trial examining the efficacy of an exercise intervention for the prevention of postpartum depression (study was conducted from January 2010 through November 2011). Participants completed questionnaires examining demographic characteristics and psychosocial variables at 6 weeks and 7 months postpartum. The Edinburgh Postnatal Depression Scale was administered at 7 months postpartum to assess depression symptomatology. Sixty-eight percent of the participants reported that they were employed at 7 months postpartum. Employment at 7 months postpartum was associated with lower depression symptomatology (as measured by the Edinburgh Postnatal Depression Scale) after controlling for condition assignment, marital status, and having other children. Among women who worked outside of the home, there were no differences between those who worked full-time versus part-time on depression symptomatology. Employment may be a protective factor for postpartum depression symptomatology; however, we cannot infer causation given this study’s cross-sectional design. Postpartum women at risk for depression who are contemplating employment should consider the possible protective effect of employment on depression.

## Introduction

Approximately 13%–19% of new mothers report depression during the postpartum period.^1,2^ Negative consequences associated with postpartum depression include poor bonding with the infant,^3^ high weight retention for the mother,^4^ difficulty taking care of the newborn,^5,6^ and a higher risk of future depression for both parents.^7^ Therefore, it is important to examine protective factors related to the onset of postpartum depression.

Research indicates that working outside of the home may be a protective factor for postpartum depression.^8,9^ A cross-sectional study among 198 first-time mothers found that having a low income, being unmarried, having less than a college education, and being unemployed were related to clinically high depression scores at 3 months postpartum.^9^ One limitation of this study is that this study only included first-time mothers, and it is unclear how the findings would generalize to mothers who have multiple children.

A similar study examined employment status and depression among 700 women who had given birth on average 7 months prior to the assessment. Findings indicated that employed participants were less likely to report depressive symptoms than those not employed.^8^ These results were maintained after controlling for race/ethnicity, age, education, marital status, family income, infant’s health status, parity, and maternal depression in the first 8 weeks postpartum. One problem with this study is that depression was assessed using only a two item questionnaire (the Patient Health Questionnaire 2 (PHQ-2)). Another study among 1,359 mothers found that being employed was a protective factor for depression during the first 2 years following childbirth.^10^

Research also indicates that among women who return to work after giving birth, returning to work later (i.e. 13–24 weeks) relates to improved postpartum mental health.^11^ However, there may be a limit to the protective effects of returning to work. In another study, greater total workloads (paid and unpaid work) were associated with poorer mental health at 12 months postpartum.^12^ Greater work spillover to home has been associated with worse mental health scores compared to those who had lower work spillover.^13^ Research also indicates that women who work part-time report fewer depressive symptoms than women who are unemployed.^14^ Additionally, new mothers who have a history of depression are almost three times more likely (31% risk) to develop postpartum depression than women who do not have a depression history.^15^ It is unclear if employment status has a protective effect on postpartum depression among women who are at an increased risk for postpartum depression. The purpose of the current study was to examine the relationship between postpartum employment and postpartum depression symptomatology.

This study was a post hoc analysis from a previously conducted randomized controlled trial. Our sample consisted of women at risk for depression (defined as having a personal or maternal history of depression), which is a population that has not been specifically examined in previous studies. We controlled for potential confounding variables in the analysis, including race/ethnicity, body mass index, education level, income level, marital status, age, antidepressant medication use, breastfeeding status, and having other children. Research indicates that postpartum depression is related to body mass index,^16^ age,^17^ race/ethnicity,^18^ and the use of antidepressant medication,^6^ and therefore, these variables were controlled for in the initial analysis. We assessed postpartum depression at 7 months postpartum using the Edinburgh Postnatal Depression Scale (EPDS). We hypothesized that women who returned to work would report lower levels of depression symptomatology based on the EPDS than those who did not return to work. We explored the relationship between working full-time versus part-time on depression symptomatology.

## Methods

### Participants

**Figure 1.**
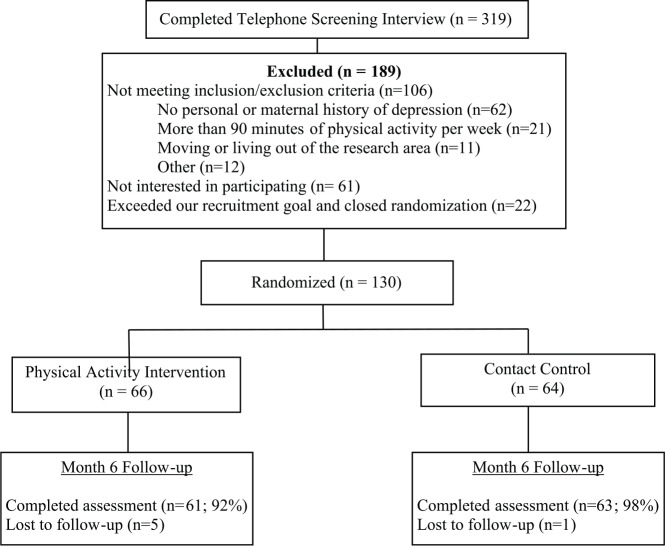
Trial flowchart.

This study was a post hoc analysis from a previously conducted randomized controlled trial. Participants (n = 124) were recruited for the overall trial during pregnancy or prior to 6 weeks postpartum from the upper Midwest in the United States through several strategies including targeted emails (the local newspaper sent emails to women between the ages of 18 and 40 who had signed up to receive emails), Craigslist (online advertising), and a local parent magazine (see Figure 1 for recruitment and retention of the overall trial). Participants were healthy postpartum women who had participated in a randomized trial examining the efficacy of an exercise intervention for the prevention of postpartum depression. Participants were between the ages of 18 and 42 and had a personal or maternal family history of depression. Exclusion criteria included the following: (1) exercising more than 60 min per week, (2) participating in another exercise-related study, (3) less than 18 years of age, (4) another household member participating in the study, (5) non-English speaker, (6) not able to walk for 30 continuous minutes prior to pregnancy, (7) pre-existing hypertension or diabetes, (8) musculoskeletal problems that may interfere with exercising, (9) exercise-induced asthma, (10) any condition that would make exercise unsafe, (11) psychiatric-related hospitalization in the past 6 months, and (12) taking medication that affects heart rate response to exercise. In order to recruit participants who were at risk for postpartum depression, eligibility criteria included a history of depression for either the participant or her mother. Participants were screened for inclusion and exclusion criteria via a telephone screening interview. Questions were based on the Physical Activity Readiness Questionnaire (PAR-Q).^19^

Eligible participants received consent forms through the mail. We randomized consented participants into the study at approximately 6 weeks postpartum, after completion of participant and healthcare provider consents and baseline questionnaires. Prior to randomization, participants completed the Structured Clinical Interview for *Diagnostic and Statistical Manual of Mental Disorders, Fourth Edition* (DSM-IV) Axis I Disorders (SCID-I) over the telephone, which has been shown to be reliable when compared to the in-person version of the SCID.^20,21^ One participant was excluded from the study due to meeting criteria for depression at the randomization session (this participant was not randomized). This study was conducted from 2009 to 2012 in the upper Midwest region in the United States, was approved by the Institutional Review Board at the University of Minnesota (#0903S61462), and all participants completed written informed consent forms. This trial is registered with clinicaltrials.gov (trial registration number is NCT00961402).

### Measures

Demographic variables, including income, marital status, and education level were assessed via a questionnaire, while race/ethnicity, age, body mass index, antidepressant use, breastfed at birth, history, or maternal history of depression (defined as ever being told they or their mother had depression or being given antidepressant medication for depression), and employment status were assessed via a telephone-based interview. Demographic questions were adapted from previous trials.^22,23^ Participants also completed the 10-item EPDS, which was administered over the telephone.^24^ The telephone-based version of the EPDS has been shown to be reliable and valid.^25^ The EPDS assesses level of depression symptomatology among postpartum women. Scores of 10 or above are considered probable depression. This scale has good specificity and sensitivity among women in the United States (78% sensitivity and 99% specificity).^26^

### Procedure

For the overall randomized trial, participants (n = 130) were randomly assigned to a telephone-based exercise intervention or a health and wellness/support contact control, both lasting 6 months. We randomized participants at 6 weeks postpartum on average, which was baseline. Based on the (SCID-I), there were no differences between participants in the two study arms on depression at 6 months (8% were depressed for each group based on the SCID). Both conditions exercised at similar rates despite the wellness/support condition not receiving exercise information (128 min of exercise per week for the exercise condition and 122 min for the wellness/support condition). We summarize the study design and results of the overall trial in more detail elsewhere.^27,28^

For the current study, participants (n = 124; six participants did not complete the six-month assessment) completed a demographic questionnaire assessing income, marital status, education level, and having other children, which was administered via mail along with the consent form. Participants mailed the consent form and demographic questionnaire back to the research assistant via a self-addressed stamped envelope. We assessed race/ethnicity during the telephone screening interview and age, body mass index, and antidepressant use at baseline (approximately 6 weeks postpartum) via a telephone-based interview. At 7 months postpartum, employment and breastfeeding statuses (i.e. if they ever breastfed) were assessed via a telephone-based interview. Participants also completed the EPDS at 7 months postpartum over the telephone.

### Data and power analysis

We used chi-square analysis to examine the relationship between the dichotomous demographic/psychosocial variables and employment status. One-way analysis of variance (ANOVA) tests examined the relationship between the continuous demographic/psychosocial variables and employment status. Logistic regression was first utilized to evaluate the relationship between employment status and postpartum depression symptomatology after controlling for several demographic/psychosocial variables (i.e. race/ethnicity, body mass index, education level, income level, marital status, age, antidepressant medication use, ever breastfed, and having other children) and condition assignment (exercise vs. wellness/support contact control condition). Previous literature guided the identification of potential confounders. We excluded potential confounders from the final analysis that were above a p-value of .15. We used r^2^ as the index for goodness of it. The above analyses were post hoc and were not included in the original study protocol. The post hoc power analysis indicated that with a sample size of 124 and an odds ratio of 3.92, we would have 91% power to detect an effect of employment status on depression symptomatology. A priori power calculations were not conducted for this specific analysis.

## Results

### Overall sample summary

The majority of the sample was Caucasian, married, college educated, and had an annual income of over US$50k (Table 1). Eighty-four percent had a personal history of depression. We randomized 66 participants to the exercise intervention, and 64 to the wellness/support contact control condition in the overall trial. Participants on average completed 10 of the 11 intervention sessions, and there were no differences between participants by study condition regarding number of sessions completed. Ninety-two percent of the exercise intervention group and 98% of the wellness/support control group completed the follow-up with no differences between the groups for follow-up rates. There were no differences between the groups on employment status.

**Table 1.** Demographic variables by employment status.

Variable	Total sample	Employed	Not employed	p-value
	(n = 124)	(n = 84)	(n = 40)	
Caucasian (%)	82.3%	84.5%	77.5%	.451
Body mass index (BMI)	28.7 (5.88)	28.9 (6.39)	28.4 (4.65)	.678
Education (%college grad)	69.4%	75.0%	57.5%	.061
Income (% over US$50,000)	58.1%	63.1%	47.5%	.121
Marital status (%married)	82.3%	84.5%	77.5%	.237
Age (average in years)	30.8 (4.92)	30.7 (5.05)	31.0 (4.68)	.753
Antidepressant use	19.4%	16.7%	25.0%	.332
Breastfed at birth	92.7%	91.7%	95.0%	.717
Have other children	70.8%	69.0%	72.5%	.834
Depression score^a^	5.87 (4.43)	5.20 (3.54)	7.28 (5.68)	.014

Standard deviations are in parentheses.

a Depression score is based on the Edinburgh Postnatal Depression Scale.

### Employment status rates

Sixty-eight percent of the participants reported employment at 7 months postpartum. There were no differences between the employment status groups on any of the demographic variables. Among those employed, 57.1% worked full-time.

### Relationship between employment status and depression symptomatology

Twenty percent of participants scored 10 or higher on the EPDS indicating probable depression. Logistic regression analyses indicated that after controlling for marital status, condition assignment, and having other children, employment status was significantly associated with higher depression symptomatology based on the EPDS (Table 2). Specifically, employed participants at 7 months postpartum were less likely to report higher depression symptomatology than non-employed women. Being single was also significantly associated with higher depression symptomatology at 7 months postpartum. Race/ethnicity, body mass index, education level, income level, age, taking an antidepressant medication, and ever breastfed were excluded from the final analysis given their p-values were above .15 in the initial analyses. Regarding overall fit, the model accounted for 34.1% of the variance. Among women who worked outside of the home, there were no differences between those who worked full-time versus part-time on depression symptomatology, r = −.022, ns.

**Table 2.** Relationship between employment status and depression.

Variable	Beta^a^	p-Value	Exp(B)	95% CI
Marital status	2.068	.001	7.907	2.429, 25.746
Condition (exercise vs. wellness)	1.697	.004	5.459	1.713, 17.402
Other children	.837	.149	2.310	.741, 7.203
Employment status	1.377	.010	3.965	1.399, 11.237

CI: confidence interval.

a Betas are logistic regression coefficients.

## Discussion

Our findings support previous research indicating that postpartum women who are employed were less likely to report higher depression symptomatology than postpartum women who are not employed.^8,9^ Based on our power analysis, our study was adequately powered to detect this effect. Our study adds to the literature by finding that the protective effect of employment on postpartum depression also occurs in women who are at an increased risk for depression. This is important given women who have a history of depression are almost three times more likely (31% risk) to develop postpartum depression than women who do not have a history of depression.^15^

The rate of employment was higher in our study (68%) compared to previous studies (19%–36%).^8,9^ Our findings are somewhat consistent with another study, which found that 80% of women employed during pregnancy returned to work by 1 year postpartum.^29^ However, it is difficult to compare employment rates with previous studies given differences in race, ethnicity, income level, education, and assessment time points between studies. Among participants who worked outside of the home, there were no significant differences between part-time versus full-time employees on depression symptomatology. This study did not have sufficient power to detect differences between part-time and full-time participants, and therefore, we cannot make definitive conclusions about this result. However, it is important to note that the correlation observed for the association between full versus part-time work and depression symptomatology was very low (r = −.022). Consequently, it is unlikely that a larger sample size would have yielded a significant relationship.

Previous research has shown that even though postpartum employment appears to relate to better mental health, certain work characteristics might detract from this favorable relationship. For example, declines in postpartum mental health relate to the following: (1) Work characteristics such as total workload;^12^ (2) higher psychological demands, lower schedule autonomy, and lower perceived control over work and family;^30^ and (3) reductions in favorable job characteristics such as job control, perceived job security, flexible start and finishing times, and provision of family-related leave including paid maternity leave.^31^ Future research should address specific employment characteristics that promote postpartum mental health.

### Limitations

This study has several limitations. First, the results are correlational, and therefore, we cannot infer causation. It is possible that participants with depression were more likely to choose not to return to work. However, none of the participants were depressed at baseline, and therefore, this seems unlikely. Another limitation is that we retrospectively assessed employment status at approximately 7 months postpartum. Third, personal and family history of depression was not clinically verified and relied only on participant self-report. Fourth, our sample consisted of mostly Caucasian, high-income, and highly educated women and it is unclear how these findings would generalize to a more diverse sample. Given the high education and income level of this sample, many of the participants may have returned to higher paying jobs, which may not generalize to socioeconomically diverse women. Additionally, our exclusion criteria were extensive, which may also limit generalizability. However, it is important to note that a majority of women were excluded due to not having a personal or maternal history of depression or engaging in over 90 min of physical activity at baseline. Also related to generalizability, all participants received some form of intervention, and therefore, it is unclear if these results would generalize to women who do not receive extra support. A final limitation is that we only assessed depression at 7 months postpartum. This is problematic given depressive symptoms may have fluctuated throughout the postpartum period.

### Future directions

There is a need for additional research that examines the relationship between employment and postpartum depression. Specifically, future studies should include longitudinal designs that examine number of hours worked, working from home, and work-related stress. It will be important for researchers to examine depression at multiple time points during the postpartum period and with a diverse sample of women. Future research should also examine the effect of type of work on depression. For example, a recent study found that women who worked from home reported a decrease in depression scores compared to those who worked outside of the home between 6 and 24 months after childbirth.^32^ Also, in this study, participants who reported higher job concerns reported increased depression over time. Future studies should examine various work factors and how they influence depression.

## Conclusion

In summary, our study indicates that employment after childbirth among a sample of women at risk for postpartum depression may be a protective factor for depression, even after controlling for multiple factors related to depression. Women may want to consider this possibility, especially those with a history of depression, when deciding whether or not to return to work after childbirth. Unlike previous studies, there was no relationship between working full-time vs. part-time and depression. Our findings are correlational and therefore, should be interpreted with caution. There is a need for long-term longitudinal studies that examine work-related factors that may be associated with depression among diverse women.
